# Current Therapeutic Modalities for the Management of Chronic Diabetic Wounds of the Foot

**DOI:** 10.1155/2023/1359537

**Published:** 2023-02-10

**Authors:** Olajumoke Arinola Oyebode, Sandy Winfield Jere, Nicolette Nadene Houreld

**Affiliations:** Laser Research Centre, Faculty of Health Sciences, University of Johannesburg, P.O. Box 17011, Doornfontein, South Africa 2028

## Abstract

Impaired wound healing is common in patients with diabetes mellitus (DM). Different therapeutic modalities including wound debridement and dressing, transcutaneous electrical nerve stimulation (TENS), nanomedicine, shockwave therapy, hyperbaric (HBOT) and topical (TOT) oxygen therapy, and photobiomodulation (PBM) have been used in the management of chronic diabetic foot ulcers (DFUs). The selection of a suitable treatment method for DFUs depends on the hosts' physiological status including the intricacy and wound type. Effective wound care is considered a critical component of chronic diabetic wound management. This review discusses the causes of diabetic wounds and current therapeutic modalities for the management of DFUs, specifically wound debridement and dressing, TENS, nanomedicine, shockwave therapy, HBOT, TOT, and PBM.

## 1. Introduction

Blood glucose is the main supplier of energy, and cellular uptake is facilitated by the pancreatic hormone insulin. Increased blood glucose (hyperglycaemia) develops as a result of deficiencies in both insulin secretion and action, affecting the metabolism of carbohydrates, lipids, and proteins. Typically, prolonged hyperglycaemia causes diabetes mellitus (DM), a group of metabolic diseases characterised by chronic high blood glucose. The severity of DM and its symptoms is affected by its type and duration [1]. According to “Classification and Diagnosis of Diabetes: Standards of Medical Care in Diabetes” (2021) [2], DM has been classified into 5 general categories, viz. (a) type 1 DM (T1DM) that develops due to autoimmune destruction of pancreatic *β*-cells leading to total insulin deficiency; (b) type 2 DM (T2DM) that develops due to reduced or inadequate insulin secretion by *β*-cells mainly accompanied with insulin resistance; (c) monogenic DM including neonatal and maturity-onset in adolescents; (d) diabetes due to other causes including cystic fibrosis and pancreatitis, and/or drug/chemical-induced; and (e) gestational diabetes that develops during pregnancy and is usually diagnosed in the second or third trimester.

In the progression of T2DM, there is a period in which the disease is undiagnosed and is estimated to last up to 10 years. In this period, increased blood glucose creates functional and structural impairments which yield macrovascular and/or microvascular impediments. Studies have reported that most of the newly diagnosed T2DM patients already have chronic impediments at the time of diagnosis [3]. According to the 10th edition of the *International Diabetes Federation (IDF) Atlas 2021* [4], DM persists as a serious public health challenge and causes a huge burden on the affected individuals and their families. Individuals with DM are at risk of developing life-threatening impediments leading to reduced quality of life and premature death. Globally, diabetes is included in the top 10 diseases with a high mortality rate and an increased need for medical care. It is estimated that one in ten adults are diabetic, and the global prevalence in adults (20–79 years) is estimated at 537 million (10.5%) [4]. By 2030, it is predicted that globally, 643 million people (11.3%) will have diabetes if there is no appropriate achievement to address the situation [4]. With this increasing trend, it is predicted that by 2045, 783 million adults (12.2%) will be affected. In 2021 alone, approximately 6.7 million adults were estimated to have died as a result of DM or its impediments [5]. Chronic DFUs are the most common impediment in uncontrolled DM, mainly due to poor glycaemic control, neuropathy, peripheral vascular disease (PVD), and inadequate foot care. It is a common cause for lower extremity amputation. These wounds usually develop in areas which encounter recurring trauma and pressure [6]. The prevention of DFUs is prioritised by identifying high-risk individuals, specifically those with peripheral neuropathy, PVD, and foot calluses and deformities. DFUs affect approximately 15% of all diabetic patients, and 15 to 20% of patients with such wounds frequently necessitate amputation [7]. This review is an attempt to reveal causes of diabetic wounds and current therapies used in the management of DFUs.

## 2. Major Causes of Diabetic Wounds

**Figure 1:**
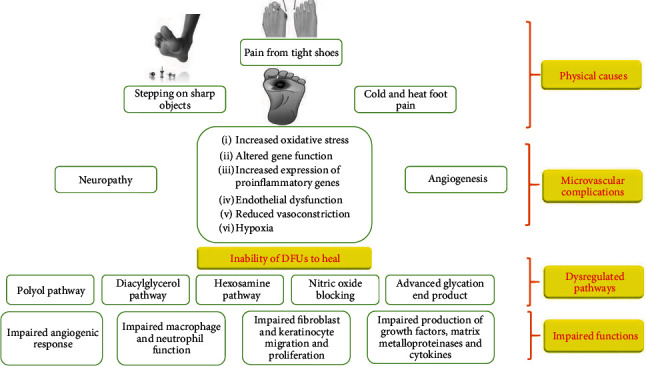
A summary of the major causes of chronic diabetic wounds.

Diabetic wounds develop due to a variety of causes, including physical trauma, microvascular complications, dysregulated pathways, and impaired systemic functions as summarised in Figure 1. However, the influencing factors for wound chronicity and lower limb amputation include neuropathy, PVD, and foot infection.

### 2.1. Neuropathy

Diabetic neuropathy is characterised by the loss of sensation in the lower extremities and is a frequent underlying cause of DFUs [4]. Approximately 26% of patients with T2DM have confirmed peripheral nerve damage at the first visit for diagnosis, and almost 50% of diabetic individuals develop diabetic neuropathy [3]. Diabetic neuropathy leads to the malfunction of peripheral nerves and is generalised as polyneuropathy [8]. Symptoms of diabetic neuropathy vary from pain and numbness in the feet and legs to complications of the urinary tract, digestive system, blood vessels, and heart. Reduced sensation to the lower extremities aggravates the development of DFUs as a result of repeated trauma and failure to detect the insult. In this way, wounds are ignored and gradually deteriorate. The treatment for diabetic neuropathy includes effective glucose control to halt its progression [7].

### 2.2. Peripheral Vascular Disease (PVD)

PVD develops as a result of the thickening of the arteries (due to build-up of deposits in the arterial linings) and includes peripheral arterial disease (PAD), coronary artery disease, and life-threatening cerebrovascular disease (CVD). PAD is one of the major underlying complications of DFUs. It is caused by the atherosclerotic narrowing of outlying arteries of the lower extremities due to systemic atherothrombosis and can cause significant long-term debility, lower extremity amputation, and death [9]. The pathologic states of DM including hyperglycaemia, dyslipidaemia, and cellular resistance to insulin promote the development and progression of PVD, mostly through disorders in the vessel wall, vascular inflammation, malfunctioning of endothelial cells, abnormal blood cells, and factors related to haemostasis. In DM, these defects are frequently established preceding the diagnosis, and their severity increases with deteriorating control of blood glucose and the period of DM [10]. Agents that lower blood glucose may have a significant influence on the development of atherosclerosis and PVD [11].

### 2.3. Foot Infection

Due to neuropathy, vascular insufficiency/PVD, and reduced neutrophil function, diabetic patients are susceptible to foot infections. Diabetic neuropathy causes loss of defensive sensations such as temperature and pain which impair the recognition of blistering, abrasions, and piercing by foreign bodies. Broken skin exposes the underlying tissues to colonization by pathogenic microorganisms resulting in wound infection. Delays in wound management and diminished natural defence mechanisms as a result of minimal function of neutrophils can lead to the infection extending to adjoining internal and deeper tissue [12]. Wound infections are the major cause of ill health in diabetic patients, and in the absence of treatment, they can lead to nontraumatic amputations. In most cases, bacteria including staphylococcal, streptococcal, and facultative anaerobes such as *Bacteroides* species contaminate DFUs, and single or multiple species of bacteria may be isolated from the infected wound [13]. Infections are usually clinically diagnosed, and their diagnoses are centred on the presence of inflammation and purulence. Diabetic wound infections are classified as mild, moderate, and severe, and osteomyelitis is a critical complication of infection, increasing the prospect of surgical amputation. The treatment for diabetic wound infection is centred on the magnitude and gravity of the infection and comorbidity [14].

## 3. Progression of Diabetic Wound Healing

The normal wound-healing process is multifaceted and dynamic resulting in reestablishment of the tissues' anatomic integrity and function. Effective wound management results in faster healing exclusive of infection and sepsis. Acute wound healing is divided into 4 phases including haemostasis, inflammation, proliferation, and remodelling. Each phase is typified by strategic molecular and cellular actions coordinated by cellular factors within the wounded tissue. In DM, wound healing tends to be slow with rapid progression to wound ulceration [15] and is mainly characterised by chronicity of the inflammatory phase, disordered angiogenesis, reduced endothelial progenitor cells, and an imbalance in the regulation of extracellular matrix (ECM) proteins [16]. In diabetic wounds, the levels of inflammatory cytokines including tumour necrosis factor-alpha (TNF-*α*), interleukin-6 (IL-6), and interleukin-1*β* (IL-1*β*) released by infiltrating cells are elevated and remain at high concentrations for longer periods resulting in a prolonged inflammatory phase. Consequently, reduced blood circulation to the lower limbs leads to a decrease in the delivery of nutrients which makes the tissues hypoxic favouring reduced cell migration and proliferation. There is also reduced collagen synthesis and increased destruction of collagen by elevated matrix metalloproteinases (MMPs) [17]. In addition, there is compromised production and function of growth factors including transforming growth factor-beta (TGF-*β*) and insulin-like growth factor-1 (IGF-1) that under normal circumstances initiate and sustain the wound-healing process [18].

## 4. Current Therapeutic Modalities for Management of DFUs

Most diabetic wounds are categorised as chronic since they are slow to heal (often taking more than 3 months) and do not partake in the normal wound-healing sequence. The loss of mobility and the high cost of treatment makes the demand for an efficient and faster wound-healing process more desirable. For diabetic patients, wound prevention and efficient wound management guidelines must be implemented to eliminate the need for limb amputations. Physical examinations are carried out to determine the extent of the wound, followed by vascular examination which covers the wound position, depth, size, and affected tissue type. In addition, the sampling of the wound edge is critical in the determination of infection. The “TIME” principle is used as a general approach in the management of chronic wounds, involving tissue debridement, infection control, moisture balance, and edges of the wound [19]. Following this assessment, other therapeutic modalities can be used to ensure effective healing. Andrews et al. [20] indicated that there is a high mortality rate in patients with DFUs in comparison with diabetic patients without foot ulcers.

**Figure 2:**
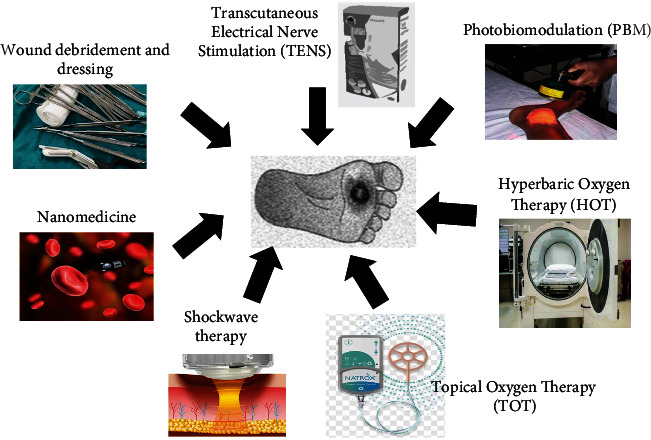
Conventional methods in the management of diabetic wounds.

The principal objective in the treatment of chronic DFUs is to achieve quick wound resolution and closure, and the choice of treatment should primarily focus on preventing lower limb amputation. This approach is carried out through 3 key steps including identification of diabetic patients at risk, treatment and care of the acutely affected foot, and avoidance of recurrence and additional impediments [21]. Currently, several conventional strategies in the management of diabetic wounds including wound debridement and dressing, TENS, nanomedicine, shockwave therapy, HBOT, and PBM are being used with limitations, including the rate and progress of healing and wound recurrence (Figure 2). The corrected irregularities in the management of diabetic wounds using these conventional methods are shown in Table 1.

### 4.1. Wound Debridement and Dressing

Nonviable tissue present on the edge of chronic DFUs hinders the wound-healing process. To facilitate chronic wound healing through all the healing stages, the wound bed should be free of nonviable tissue, well vascularized, moist, and uninfected. Wound debridement is a key procedure in wound management mostly used to correct cellular and molecular anomalies and set up the wound bed for reepithelialisation. This technique involves keeping the wound site free of nonviable callus, necrotic, senescent, and fibrous tissue and biofilm to encourage wound healing [22].

Biofilm, with bacterial or fungal (or both) colonies that inhabit the wound surface, is a major barrier to wound healing and is frequently resistant to antibiotics and other treatment methods [23]. The formation of a biofilm is a periodic process in which microbial cells attach to the wound surface and subsequently produce an extracellular matrix made up of proteins, polysaccharides, and DNA which favour a stronger bond to the wound site [24]. Recently, wound debridement has focused on biofilm reduction and eradication through topical therapies, application of negative pressure, and ultrasound (US) therapy [25].

A low-frequency US device is mainly used to debride and clean the wound. The procedure is painless and is beneficial in getting rid of the biofilm present at the wound site. However, Breuing et al. [26] noted that the use of US is less effective in venous stasis and diabetes-induced chronic wounds than in chronic wounds of pressure, surgical, and arterial insufficiency and that multiple applications are required to disrupt the biofilm and advance wound healing. The use of other debridement techniques including, but not limited to, sharp surgical, autolytic, mechanical, hydrotherapy and enzymatic have been reported to be effective in facilitating wound healing [27, 28].

Wound dressings help facilitate the debridement process as they further eradicate dead tissue, inhibit bacterial growth, regulate exudate, and control fluid balance. This procedure plays a key role in effective wound healing, and it involves maintaining a moist and infection-free wound environment [29]. However, the role of dressing as a sole wound management modality is limited. Wound debridement and dressing with the application of antimicrobial creams could be used together to promote wound healing. When combined with hydrogels produced using two polymers (chitosan and cellulose), debridement has been reported to be effective in wound healing [30].

Debridement using enzymes such as collagenase has been seen to effectively remove dead tissue without harming healthy tissue and contributes to the formation of granulation tissue and ultimately reepithelialisation. Collagenase has been reported to stimulate migration and proliferation of fibroblasts and keratinocytes through the release of stimulatory peptide fragments. Although some scientists may be concerned that debridement could cause a portal for the entry of bacteria since the skin is exposed during the treatment, studies have shown that the rate of infection does not increase after debridement [31]. Recently, larva debridement therapy, a biotherapy that involves the presentation of disinfected maggots (fly *larvae*) into the nonhealing wound, has also been successfully implemented to induce healing in chronic DFUs [32, 33]. Complications of wound debridement range from local irritation to heavy bleeding, with medium to severe pain varying on the type of debridement technique chosen [34–36].

### 4.2. Transcutaneous Electrical Nerve Stimulation (TENS)

The noninvasive and superficial application of electrical stimulation (ES) to treat chronic wounds is known as TENS. Clinical and animal studies have reported on the benefits of ES to wound healing [37, 38]. Although TENS is painless, research has shown that its application causes an increase in local wound temperature and improved blood flow at the wound site [39, 40].

The mechanism of action of TENS includes an increase in wound epithelialisation, wound contraction, and increased angiogenesis [41]. The application of electrical impulses to chronic wounds has also been reported to elicit an increase in the proliferation and migration of fibroblasts, as well as an increase in growth factor expression and phagocytosis [42, 43]. In addition, TENS facilitates the wound-healing process by increasing protein synthesis in fibroblasts, minimising oedema, and limiting infection by improving blood flow to the wound [41]. A study by Liebano et al. [44] proposed a low frequency of 2 Hz as the preferred frequency for optimal wound healing. In the study, TENS electrodes were attached to the sides of the wound surface for 15 minutes per day for 5 days. The applied low-frequency TENS treatment was shown to be effective in improving the condition of the wound.

A recent study also reported that TENS induces wound healing by increasing the action of growth factors such as epidermal growth factor (EGF), platelet-derived growth factor-A (PDGF-A), and fibroblast growth factor-2 (FGF-2) at the wound site [43]. An *in vivo* study examined the comparative effects of wound dressing with physiological saline and topical povidone iodine application as well as TENS on the expression of proinflammatory cytokines. The study suggested that TENS application had an anti-inflammatory effect through the inhibition of proinflammatory cytokine (IL-1*β*, TNF-*α*, and IL-6) activity. However, it was noted that a combination of stimulation parameters and wound type makes TENS less attractive for the management of wounds [45].

### 4.3. Nanomedicine

The emergence of techniques and products related to nanoparticles (NPs) has offered promising results for different diseases. In chronic diabetic wounds, nanoparticles promote healing by delivering bioactive molecules including, but not limited to, cytokines/growth factors, peptides, genes and stem cells, and nonbioactive molecules including, but not limited to oxygen, metal ions and nitric oxide. Recently, the use of nanoparticle matrices has shown positive effects on wound healing through the delivery of proliferative factors and elevation of angiogenesis and collagen synthesis. Typically, nanotechnology enhances wound healing through all the phases of healing including the regulation of inflammation, stimulation of cell regeneration, and skin reepithelialisation [46, 47]. NPs are a basic constituent of the nanostructure with a distinctive size and qualities. In chronic diabetic wounds, the NPs used for wound healing largely include metallic and nonmetallic nanomaterials. Nanotechnology has critically contributed to the achievement of high drug concentrations within the wound with reduced side effects when compared to conventional drug delivery techniques. The two main principles of NPs used in wound healing are based on the direct effect of NPs on wound healing and its use as a delivery agent [48].

The problem encountered in using conventional treatment methods has boosted research in nanotechnology. This is mainly due to the small molecular size and numerous functions that NPs possess. Considering their ability to transport drugs to target areas, reduce drug side effects, deliver constant drug release, and improve on the stability of drug action, NPs have shown to be convenient and efficient for chronic diabetic wound healing [49]. NPs including gold, curcumin, chitosan, and silver have been extensively used in studies related to diabetic wound dressings, and amongst these NPs, there is mounting research on physical relationship, biocompatibility, and biodegradability of biopolymers and normal skin. Several researchers have studied the effect of nanotechnology in diabetic wound healing. Gainza et al. used rhEGF-loaded Poly-Lactic-co-Glycolic-Acid- (PLGA-) Alginate microspheres (MS) for the treatment of diabetic wounds. They demonstrated that rhEGF-loaded MS effectively promote wound healing, suggesting its possible use in the treatment of DFU [50]. In another study, Liu et al. used a thermos-sensitive hydrogel that contained a nanodrug in the form of gelatin microspheres (GMs) designed to transport curcumin (Cur) to the wounded tissue. Their results showed a significant increase in promoting cell migration and skin wound healing [51]. Recently, Sonamuthu et al. demonstrated the effective inactivation of MMPs and inhibition of microbial infection when they evaluated the inactivation of MMP and reduction of microbial infections using a combined curative effect of metal-chelating dipeptide (L-carnosine) and curcumin on the infected diabetic ulcer [52]. Haque et al. described different *in vitro* and *in vivo* clinical nanotechnology-based treatment methods that could be considered for use in the clinical treatment of DFUs [53].

Some NPs including silver and gold are important for wound dressings as they possess antibacterial action and reduce side effects [54]. However, variable preparation processes and the lack of biosafety evaluation systems create a challenge in the use of nanotechnology and NPs in medicine. Furthermore, while nanotechnology is being employed in various treatment trials, difficulties in understanding the NPs' biocompatibility, biodegradability, time for drug release, stability and integrity with biomacromolecules, and their choice still need to be critically put into consideration for research [49].

### 4.4. Shockwave Therapy (SWT)

Recently, SWT has been seen to significantly advance the healing of soft tissue and has demonstrated positive outcomes in DFUs [55]. The use of extracorporeal SWT (ESWT) has been reported to be effective and safe in the treatment of chronic wounds in both short- and long-term therapies. A study by Wang et al. [56] reported on the efficacy of ESWT treatment of DFUs. In the study, 67 patients received ESWT (500 shocks at 4 Hz; equivalent to 0.11 mJ/mm^2^ energy flux density) to the affected foot twice a week for 3 weeks for a total of six treatments. Although their study showed the success of ESWT application in chronic diabetic and nondiabetic foot ulcers, the effects decreased with years after treatment. A more recent study investigated the effect of ESWT (150–500 impulses, 5 Hz, 0.1 mJ/mm^2^) on macrophage activity in chronic wounds; effects were studied before and two weeks after ESWT application [57]. The results of the study showed that macrophage ERK activity was enhanced, suggesting that the application of ESWT is important for effective macrophage function linked with wound healing.

Although there is insufficient evidence to justify the routine use of SWT in wound healing, this therapeutic method has shown that it is able to advance the wound-healing process in DM [58]. SWT is specified for diabetic adults with chronic DFUs that have been present for 30 or more days, and common adverse events associated with this treatment methodology include bruising, pain, numbness, nausea, migraine headaches, syncope, cellulitis, wound infection, fever, and osteomyelitis [59]. Understanding and addressing the underlying conditions of a diabetic wound make treatment more effective.

### 4.5. Hyperbaric Oxygen Therapy (HBOT) and Topical Oxygen Therapy (TOT)

DM is strongly linked with reduced microcapillary perfusion, tissue hypoxia, and neuronal damage. For over 20 years, HBOT is known to be an effective treatment for chronic DFUs by improving wound tissue oxygenation, thereby reducing hypoxia, increasing perfusion, regulating inflammatory cytokines, decreasing oedema, stimulating proliferation of fibroblasts and collagen synthesis, and promoting neoangiogenesis. This observation has made HBOT an advantageous additional method of the treatment in chronic DFUs [60]. Studies have shown that the use of HBOT in the treatment of DFUs positively affects the wound-healing process and reduces the possibility of amputation. It has been suggested that HBOT has increased the survival rate of diabetic patients with DFUs. Some studies have suggested that HBOT could be applicable as an adjunct treatment method in chronic DFUs; however, incomplete scientific validation of its efficacy and safety limits its use [61–64]. For the past years, TOT has been disregarded due to an unproven treatment methodology deprived of hypothetical and experimental confirmation to back its usage. Recent findings show that TOT significantly increases oxygen concentration within the wound base and hastens the wound-healing process and is a viable treatment option in diabetic patients with DFUs [65]. TOT is the topical administration of oxygen to the wound by constant diffusion or pressurised systems. However, it is important that TOT, as any other wound therapeutic method, is managed and administered in combination with ideal wound nursing. In addition, not all TOT-treated wounds will heal as TOT is not suitable for all types of wounds [66].

### 4.6. Photobiomodulation (PBM)

Progress in technology and increased knowledge in the science of photonics and biophotonics have led to the effective use of light in the treatment of DFUs [67]. Numerous studies have demonstrated the therapeutic benefits of PBM, previously known as low-level laser (light) therapy (LLLT), on diabetic wound healing [68]. PBM was first discovered in 1968 when Mester unexpectedly saw that exposure to low-powered laser light could stimulate hair growth in mice. He later proceeded to investigate the beneficial effects of low-powered lasers on skin ulcers [69–71]. Since then, lasers, light-emitting diodes (LEDs), and broadband light-using filters have been used in both *in vitro* and *in vivo* studies [72, 73].

PBM is considered as an effective therapeutic method in wound healing when used with the correct treatment parameters [74]. PBM works through the application of light (using different light sources) to affected cells or tissue, and the absorbed light (photon) energy excites cellular molecules and atoms without causing any significant increase in temperature [75]. The ease of application of PBM and its effect on alleviating pain and reducing infection makes it a more favourable therapy for healing DFUs, thereby increasing the quality of life of diabetic patients [76]. PBM alleviates the inflammatory phase in chronic wounds and favours angiogenesis and synthesis and organisation of the ECM [77]. It is noninvasive and offers a pain-free technique of wound treatment, and the parameters used in PBM include energy density (ranging from 0.04 to 30 J/cm^2^), wavelength (ranging from 400 to 1100 nm), and application time [78]. The penetration of light through the tissue varies with wavelength. Red light has a deeper penetration depth than blue, green, violet, and yellow light. Invisible infrared and near infrared light penetrates deeper than visible red light [79].

A study investigated the therapeutic effects of blue or red LEDs on wound healing in an ischemia-disturbed rodent model [80]. The results showed that LED improved early wound healing irrespective of wavelength by enhancing angiogenesis in a noninvasive (i.e., the light only penetrates the skin but does not tear the skin surface) and cost-effective manner. In clinical trials, PBM has shown stimulatory effects in diabetic wound healing. PBM elicits increased collagen and ECM synthesis, decreased MMPs, and increased growth factor production as well as decreased activity of inflammatory cytokines [67]. There have been no reported side effects with the use of PBM. In addition, blue light (405 and 470 nm) has been found to produce antibacterial effects in wounds [81, 82]. However, the dosage and the effects of PBM at the tissue, cellular, and molecular levels are indecisive, requiring more research [83, 84].

## 5. Future Perspectives and Challenges

Chronic DFUs remain a challenging health problem in the 21^st^ century, but the good news is that important therapies are being developed for the management of these wounds. For effective wound management in diabetes, hyperglycaemia needs to be properly controlled. Aggressive and targeted combination therapies for wounds should be rigorously implemented to minimise the risk of foot amputation. There is no end to therapeutic approaches in managing diabetic wounds, as no single therapy is without contraindications. The use of PBM therapy is painless and generates no thermal effect, but the main challenge for developing PBM-based techniques as a standard treatment option is defining an exact dose-option for each wound. With new technologies revolutionising treatment therapies, an effective therapy is not far ahead. More randomised clinical trials (large sample size) and extensive research leading to the understanding of the mechanism of action of the therapies contributing to diabetic wound healing are encouraged.

## Figures and Tables

**Table 1:** Conventional methods and the corrected anomalies in the management of diabetic wounds.

Therapeutic modality	Type of procedure	Facilitated stage of wound healing	Corrected irregularities
Wound debridement and dressing (ultrasound therapy)	Invasive	All	Increases cellular and molecular activity; encourages reepithelialisation; removes callus, necrotic, senescent, and fibrous tissues; removes biofilm
Transcutaneous electrical nerve stimulation (TENS)	Noninvasive	All	Increases wound epithelialisation, contraction, and angiogenesis; increases fibroblast cell proliferation and migration; increases expression of growth factors and macrophage function (phagocytosis); increases protein synthesis; minimises oedema; limits infection
Nanomedicine	Noninvasive	All	Increases cell proliferation, elevates angiogenesis and collagen synthesis, and stimulates cell regeneration and skin reepithelialisation
Shockwave therapy (SWT)	Noninvasive	All	Increases neoangiogenesis and proliferation and reduces inflammatory effects
Hyperbaric oxygen therapy (HBOT)	Noninvasive	All	Reduces wounded tissue hypoxia, increases perfusion, regulates inflammatory cytokines, and decreases oedema. Stimulates fibroblast proliferation, increases collagen synthesis, and promotes neoangiogenesis
Topical oxygen therapy (TOT)	Noninvasive	All	Reduces wounded tissue hypoxia, increases the release growth factors, promotes neoangiogenesis, and promotes wound healing
Photobiomodulation (PBM)	Noninvasive	All	Lessens the inflammatory phase, increases angiogenesis, improves blood flow, improves synthesis and organisation of the extracellular matrix, and reduces pain and infection

## Data Availability

No underlying data was collected or produced in this study.
